# Dehydration-Induced WRKY Transcriptional Factor MfWRKY70 of *Myrothamnus flabellifolia* Enhanced Drought and Salinity Tolerance in *Arabidopsis*

**DOI:** 10.3390/biom11020327

**Published:** 2021-02-22

**Authors:** Xiang-Ying Xiang, Jia Chen, Wen-Xin Xu, Jia-Rui Qiu, Li Song, Jia-Tong Wang, Rong Tang, Duoer Chen, Cai-Zhong Jiang, Zhuo Huang

**Affiliations:** 1 College of Landscape Architecture, Sichuan Agricultural University, Chengdu 611130, China; xiangxiangying@stu.sicau.edu.cn (X.-Y.X.); chenjia@stu.sicau.edu.cn (J.C.); xuwenxin@stu.sicau.edu.cn (W.-X.X.); qiujiarui@stu.sicau.edu.cn (J.-R.Q.); songli@stu.sicau.edu.cn (L.S.); wangjiatong@stu.sicau.edu.cn (J.-T.W.); tangrong661@gmail.com (R.T.); chenduoerr@gmail.com (D.C.); 2 Department of Plant Sciences, University of California Davis, Davis, CA 95616, USA; caizhong.jiang@usda.gov; 3 Crops Pathology and Genetics Research Unit, United States Department of Agriculture, Agricultural Research Service, Davis, CA 95616, USA

**Keywords:** *Myrothamnus flabellifolia*, resurrection plant, drought tolerance, abiotic stress, WRKY transcription factor

## Abstract

The resurrection plants *Myrothamnus flabellifolia* can survive long term severe drought and desiccation conditions and soon recover after rewatering. However, few genes related to such excellent drought tolerance and underlying molecular mechanism have been excavated. WRKY transcription factors play critical roles in biotic and abiotic stress signaling, in which WRKY70 functions as a positive regulator in biotic stress response but a negative regulator in abiotic stress signaling in *Arabidopsis* and some other plant species. In the present study, the functions of a dehydration-induced *MfWRKY70* of *M. flabellifolia* participating was investigated in the model plant *Arabidopsis*. Our results indicated that MfWRKY70 was localized in the nucleus and could significantly increase tolerance to drought, osmotic, and salinity stresses by promoting root growth and water retention, as well as enhancing the antioxidant enzyme system and maintaining reactive oxygen species (ROS) homeostasis and membrane-lipid stability under stressful conditions. Moreover, the expression of stress-associated genes (*P5CS*, *NCED3* and *RD29A*) was positively regulated in the overexpression of *MfWRKY70 Arabidopsis.* We proposed that MfWRKY70 may function as a positive regulator for abiotic stress responses and can be considered as a potential gene for improvement of drought and salinity tolerance in plants.

## 1. Introduction

Plants in nature were exposed to changing environmental conditions and subjected to a variety of biotic and abiotic stresses. To increase their probability of survival, plants have to adapt to these hostile environments through evolutionary and regulatory mechanisms [1,2]. As the major abiotic stresses, drought and high salinity detrimentally impact the growth, development, and productivity of plants, even causing serious loss of agricultural yield [3,4]. Therefore, it is of great significance to discover the genes related to stress tolerance and the underlying molecular mechanism that are essential for the genetic improvement of plant stress tolerance and the sustainable development of human society.

WRKY transcription factors (WRKYs), as a large family of plant transcription factors (TF), participate in a variety of biological processes in plants, including root growth, the quality of blossom clusters, senescence of leaf, fruit maturation, and resistance to pathogens [5,6,7,8]. WRKYs are typically composed of two structures, including the highly conserved WRKYGQK heptapeptide called WRKY domain at its N-terminus and the C-terminal zinc finger motif [9,10]. WRKY domain can specifically bind on *cis*-acting elements known as W-box (C/T)TGAC(C/T), as well as RAV1A and WLS element, indicating that they have different binding modes and diversified regulation of the downstream target gene [11,12]. In recent years, increasing pieces of evidence proved that TFs serve as critical roles in biotic and abiotic stress signaling [13,14]. Some WRKY transcription factors were particularly induced by a combination of drought and cold stress treatments or drought and heat shock stress treatments simultaneously [15,16]. Overexpression of *TaWRKY33* in *Arabidopsis* and wheat reduced the leaf water loss rate and enhanced salinity tolerance [17,18]. Heterologous overexpression of *PbrWRKY53* could enhance the drought stress tolerance of tobacco and *Pyrus ussuriensis* [19]. Thus, WRKYs are considered a reservoir for the discovery of genes related to stress tolerance.

As the only woody resurrection plants, angiosperm *Myrothamnus flabellifolia,* known as homoiochlorophyllous desiccation-tolerant plants (HDTs), could rapidly reactivate after a dehydration rate of up to 95%; besides, physiological functions could restore normal operation to as it was before desiccation [20,21,22]. Although the morphological characteristics of *Myrothamnus flabellifolia* have been well studied, few genes of *M. flabellifolia* related to desiccation tolerance were characterized and the underlying molecular mechanisms remain unclear. WRKY70 belongs to the group III of the WRKY family with one WRKY domain and CCHC zinc finger motif [9]. Previous studies showed that WRKY70 was involved in the regulation of leaf senescence [23] and immune responses [24,25]. Additionally, the negative roles of WRKY70 in responding to abiotic stress were also revealed. In *Arabidopsis*, *AtWRKY70* functions as a negative regulator in abiotic stress signaling, and similar results for WRKY70 were also obtained in the tomato [26,27]. Repressing expression of *PsnWRKY70* could enhance salinity tolerance in *P. simonii × P. nigra* [28]. Previous transcriptome analysis of *M. flabellifolia* showed that *MfWRKY70*, a homologue to *AtWRKY70*, was significantly up-regulated at the early dehydration stage [29], suggesting that it may play some roles during dehydration. In this study, the *MfWRKY70* was cloned and its potential functions involved in drought and salinity tolerance were investigated in *Arabidopsis*, and the underlying mechanism was preliminarily studied.

## 2. Materials and Methods

### 2.1. Plant Materials and Stress Treatments

The *Arabidopsis* ecotype *Columbia* was used in this study and conserved by our lab. Plants were cultivated with a mixture of soil and vermiculite (1:1, *v*/*v*), raised in a growth room at 24 °C (day)/22 °C (night), 75% relative humidity and approximately 100 µM photons m^–2^ s^–1^ with a 16-h light/8-h dark photoperiod. To ensure uniform number of plants in similar status were used for further growth and analysis, seeds of *Arabidopsis* (transgenic lines and wild type) were surface-sterilized with a 1:1 diluted bleaching agent, sown on 1/2 MS (Murashige & Skoog) medium after being washed by sterilized deionized water three times, then vernalized (4 °C) for 48 h and grown in 16-h light/8-h dark (24/22 °C) conditions. Ten days later, seedlings were transplanted to plastic pots containing same weight of soil and vermiculite (1:1, *v*/*v*) and placed in the growth chamber.

For seedling stress treatments, surface-sterilized seeds of WT and transgenic *Arabidopsis* were sown on 1/2 MS medium with mannitol (0, 200, 250, or 300 mM) and NaCl (0,50, 100, or 150 mM). Before adult stage stress treatments, WT and transgenic lines were grown in the growth chamber under the same temperature (22 °C), relative humidity (75%), and light intensity (150 μmol/m^2^·s), and normally watered for four weeks. For salt treatment, the pots containing the same number (25 strains) of plants were well watered and then transferred into trays (eight pots per tray) containing the same volume of 300 mM NaCl solution. For drought treatment, the same numbers (3 strains) of plants in each pot were well-watered to keep the same initial soil water content. The watering was then withheld for three weeks, then the plants were well rewatered. During treatments, all plants were placed in the growth chamber under the same conditions as described above.

### 2.2. Cloning and Bioinformatic Analysis of MfWRKY70

Total RNA was extracted from the leaves of *M. flabellifolia* using a Plant Total RNA Isolation Kit (TINAGENE Co., Beijing, China). The first-strand cDNA was synthesized by using a Reverse Transcriptase kit (Takara Bio, Dalian, China) according to the instructions of the kit. According to uniGene sequence corresponding to *MfWRKY70*, specific forward and reverse primers were designed by Primer Premier 5.0 with additional *Nco* I and *Spe* I restriction sites, respectively. After being purified, amplified PCR products were cloned into a pEasy-T1 Simple vector (TransGen Biotech, Beijing, China) and sequenced.

Homologous proteins of MfWRKY70 from different species were selected using BLASTP of NCBI (https://blast.ncbi.nlm.nih.gov/Blast.cgi, accessed on 15 November 2020). Multiple sequence alignments between WRKY70s were analyzed using DNAMAN (version 5.2.2, LynnonBiosoft, San Ramon, CA, America). Phylogenetic tree analysis was obtained using MEGA 5.0 (MEGA, Auckland City, New Zealand) [30].

### 2.3. Subcellular Localization of MfWRKY70

Prediction of protein localization using the web-based tool (https://psort.hgc.jp/, accessed on 20 October 2019). *MfWRKY70* was cloned into a pHB-YFP vector fused with *Yellow fluorescent protein* (*YFP*). Sequence-verified recombinant plasmids 35S::MfWRKY70-YFP and 35S::YFP control vector were respectively transferred into *Agrobacterium tumefaciens*, then injected into the four weeks old tobacco (*benthamiana*) cells. After 16 h dark treatment and two days of cultivation, the *YFP* expression in tobacco cells was observed with a confocal laser scanning microscopy (Nikon, Tokyo, Japan).

### 2.4. Vector Construction and Generation of Transgenic Lines

Primers containing *Nco*yⅠ (forword) and *Spe*Ⅰ (reverse) enzyme sites were designed to add corresponding enzyme cut sits on *MfWRKY70*. The double-digested and purified amplicons were inserted into digested linear pGSA1403 by T4 DNA Ligase. The resulted 35::pGSA1403-MfWRKY70 vector was transformed into *A. tumefaciens* strains LBA4404, and the floral-dip transformation method was used to transform *Arabidopsis* [31]. T_0_ seeds were selected in 1/2 MS medium with 50 mg/L kanamycin. The selfing offsprings were further checked by PCR and three homozygous positive T_3_ lines were randomly selected for further analysis.

### 2.5. Water Loss Rate

About 0.5 g rosette leaves from same positions of four-week-old WT and transgenic plants were sampled for water loss rate analysis. Leaves were placed on filter paper in an experimental bench at room temperature (~25 °C, relative humidity 60%), and weighed at 0.5 h, 1 h, 1.5 h, 2 h, 3 h, 4 h, 5 h, 6 h, and 7 h. The water loss percentage was then calculated. Three biological replicates for each line were performed.

### 2.6. Stomatal Aperture Analysis

To investigate the change in stomatal operating of transgenic and wide-type *Arabidopsis* induced by osmotic stress and ABA, rosette leaves excised from four-week-old WT and transgenic *Arabidopsis* were floated on the MES-KCl solution (50 mM KCl, 0.1 mM CaCl_2_, and 10 mM MES, pH = 6.15). Leaves were exposed to light for 2.5 h to induce stomatal opening and were then transferred into solution with 300 mM mannitol and 20 μM ABA, respectively. Leaves treated by MES-KCl solution were used as control. After 2 h incubation, optical microscopy (DP80, Olympus, Japan) was used to observe stomatal aperture immediately. A length/width ratio of about 100 stomata was measured for stomatal aperture analysis. Three biological replicates for each line were performed.

### 2.7. Physiological Measurements

A hydrogen peroxide assay kit and an inhibition and produce superoxide anion assay kit (Nanjing Jiancheng) were used to measure the reactive oxygen species (ROS) level (H_2_O_2_ and O^2−^) by following the operating instructions. Histochemical staining using nitroblue tetrazolium (NBT) and 3,3′-diaminobenzidine (DAB) was performed as described previously [32]. Ten leaves of the same parts were taken from three individuals of WT or transgenic lines and used for staining. The malondialdehyde (MDA) content was measured flowing the thiobarbituric acid (TBA) method [33]. The activity of SOD was assayed by the NBT photoreduction method as described [34]. The activities of peroxidas (POD) and catalas (CAT) were determined by the methods reported by Zheng et al. [35]. Three biological replicates and three technical replicates were executed.

### 2.8. Reverse Transcription PCR (RT-PCR) and Quantitative Real-Time PCR (qRT-PCR)

Leaves of four-week-old seedlings were subjected to treatments of salinity stress or simulating drought stress with 300 mM NaCl or 10% PEG-6000 for three days and seven days, respectively. RNA of treated leaves were extracted by the Plant RNA Kit (Omega Bio-tek, Norcross, GA, United States) for expression analyses of stress-associated genes (*P5CS*, *NCED3*, and *RD29A*) in transgenic and WT *Arabidopsis*. Reverse transcription was conducted using reverse transcriptase Uscript II (Innovagene biotech, Hunan, China). The qRT-PCR was conducted with 2 × SYBRGreen qPCR Mix (Innovagene biotech, Hunan, China) following the manual protocol. *AtActin2* was used as internal controls. The relative expression levels were determined using the method of 2 ^−∆∆CT^ [36]. Three biological replicates and three technical replicates were conducted. Primers were listed in Supplementary Table S1.

### 2.9. Statistical Analyses

The statistical analyses were performed by Student’s *t*-test in SPSS (version 23.0, IBM Corporation, Chicago, IL, USA).

## 3. Results

### 3.1. Isolation and Characterization of MfWRKY70

**Figure 1:**
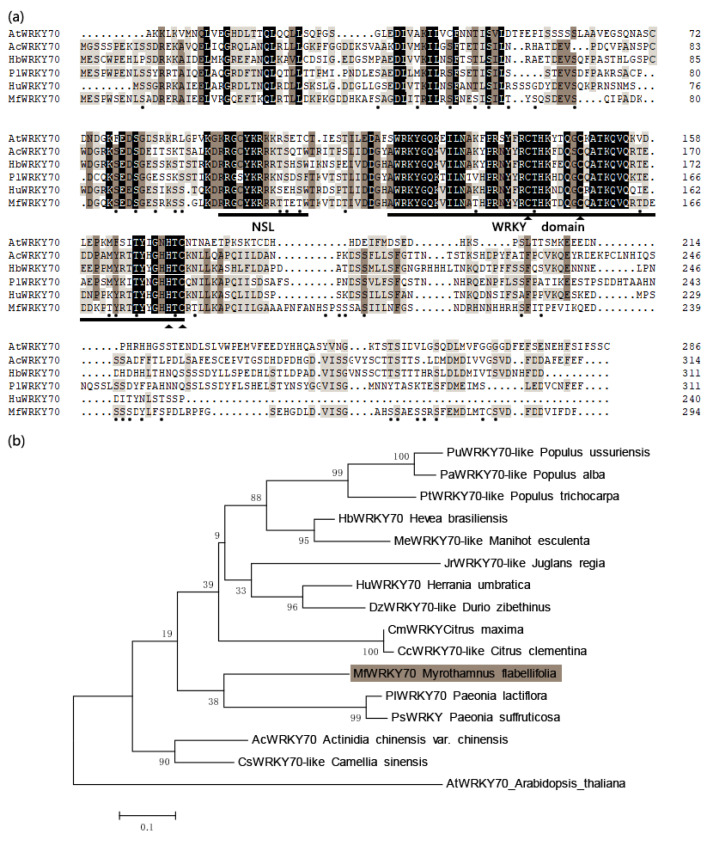
Sequence analysis and phylogenetic analysis of MfWRKY70. (**a**) Multiple alignment of deduced amino acid sequences; identical and similar amino acids are shaded in black and brown, the characteristic site of WRKY protein zinc finger structure is indicated by the arrowhead; phosphorylation sites are indicated by dots. (**b**) Phylogenetic tree constructed using the neighbor-joining method. MfWRKY70 is shaded. The GenBank accession numbers and corresponding species are listed in Supplementary Table S2.

The full length of coding region of *MfWRKY70* cDNA was cloned from *M. flabellifolia* by PCR amplification. It is 885 bp long and encodes a putative protein of 294 amino acid residues (Figure 1a). The protein has a calculated isoelectric point of 6.11 and a deduced molecular weight of 33.4 kDa. As revealed by multiple sequence alignment, MfWRKY70 was most homologous to PlWRKY70 of *Paeonia lactiflora* and AcWRKY70 of *Actinidia chinensis* (Figure 1b). Besides, a nuclear localization sequence (NLS) “RRGCYKRRRTTETW” was predicted in the N-terminal of the conserved WRKY domain. The predicted MfWRKY70 consists of a characteristic WRKY domain, containing both the oligopeptide WRKYGQK and C-X7-CX23-H-X-C (a putative zinc finger motif) (Figure 1a). Based on this, MfWRKY70 was classified as a Group III member of the WRKY transcription factor family. Moreover, some putative phosphorylation sites could be predicted (Figure 1).

### 3.2. Subcellular Localization of MfWRKY70

**Figure 2:**
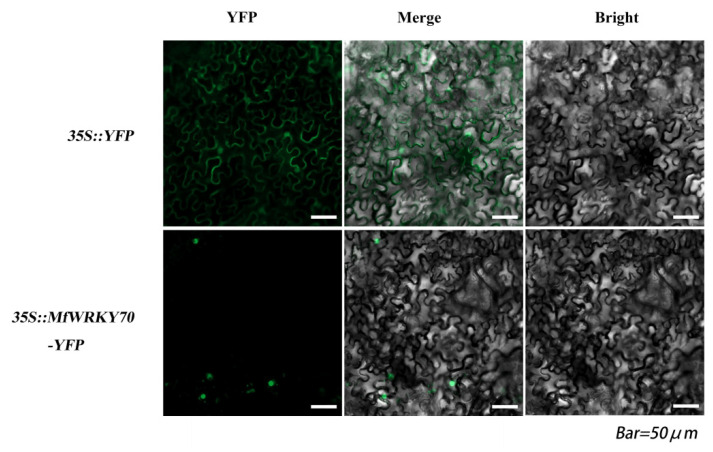
Subcellular localization of MfWRKY70-YFP fusion proteins. Fluorescence of yellow fluorescent protein (YFP) was detected in the nucleus of tobacco cells expressing MfWRKY70-YFP, and tobacco cells transformed with 35S::YFP was used as a control (upper lane).

In order to examine the subcellular localization of the MfWRKY70 protein, a vector 35S::MfWRKY70-YFP was constructed for transient expression of MfWRKY70 in tobacco. Observation using laser confocal microscopy showed that the fluorescence was detected throughout the cytoplasm and nucleus in the 35S::YFP construct, whereas the fluorescence from the 35S::MfWRKY70-YFP fusion proteins was specifically found in the nucleus (Figure 2).

### 3.3. Heterologous Expression of MfWRKY70 Improved Salt and Osmotic Tolerance in Arabidopsis

**Figure 3:**
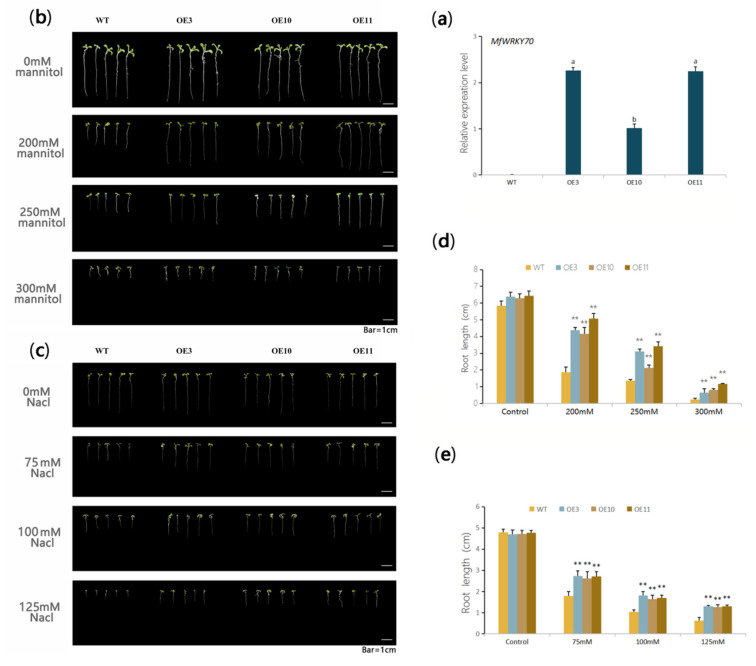
Phenotypic analysis of overexpression (OE) lines and wild type (WT) upon osmotic and salinity stresses at the seedling stage. (**a**) Relative expression levels of *MfWRKY70* in OE lines measured by qRT-PCR. Data are presented as mean and SD values of three technical and three biological replicates. Different letters above the columns indicated that the expression levels are significantly different from each other at *p* < 0.05 (LSD multiple comparison test after ANOVA). (**b**,**c**) Morphology of OE lines and WT seedlings grown on medium containing different amounts of mannitol and NaCl for one week. (**d**,**e**) Root length of one-week-old OE lines and WT seedlings containing different amounts of mannitol and NaCl, respectively. Data are presented as mean and SD values of three independent experiments. Asterisks indicate significant difference (** *p* < 0.01, by Student’s *t*-test) comparing to WT.

To determine whether manipulation of *MfWRKY70* expression would affect the growth and abiotic stress tolerance, a binary vector pGSA1403-*MfWRKY70* was constructed and used to generated transgenic lines of *MfWRKY70* under control of the CaMV 35 S promoter. We used selective media with Kanamycin and PCR to screen positive transgenic lines, and three homozygous positive lines, OE-3, OE-10 and OE-11, were randomly selected for further investigation. The real-time quantitative PCR (qPCR) analysis showed that *MfWRKY70* was expressed in all three lines, in which the expression level in OE-10 was significantly lower than in OE3 and OE11 (Figure 3a).

To further investigate the roles of *MfWRKY70* in abiotic stress tolerance, stress treatments and subsequent phenotyping were performed. At seedling stage, the root length was not significantly different between transgenic lines and WT under routine conditions (seedlings grown vertically on 1/2 MS medium for seven days). However, under the presence of 200 mM, 250 mM, and 300 mM mannitol, the root growth of WT plants was inhibited and was remarkably shorter than those of the three transgenic lines (Figure 3b). Root growth under 1/2 MS medium containing different concentrations of NaCl (50 mM, 100 mM, and 150 mM) also showed a similar result (Figure 3c).

**Figure 4:**
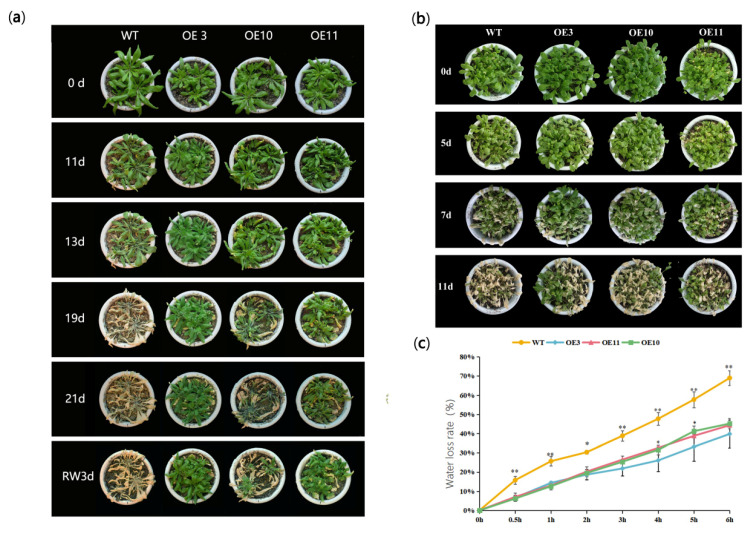
Analysis of OE lines and wild type (WT) upon drought and salinity stress at the adult stage. (**a**,**b**) Morphology of OE lines and WT plants growing under drought and salinity treatment. (**c**) Water loss rates of leaves in vitro measured at room-temperature of 25 °C. Data are presented as mean and SD values of three independent experiments. Asterisks indicate significant differences (* *p* < 0.05, ** *p* < 0.01, by Student’s *t*-test) compared to WT.

For adult plant evaluation, natural drought and NaCl treatments were applied to four-week-old WT and transgenic lines grown in soil. Before the treatment, there was no significant morphological difference between WT and transgenic lines. Stop watering for eleven days, leaves of WT were withered and chlorotic, however, almost all leaves of transgenic lines stayed green (Figure 4a). Twenty-one days after withholding (DAW) watering, WT plants were completely withered. In contrast, the wilting extent of transgenic lines, especially OE-3 and OE-11, was significantly lower, which maintained normal growth and development (Figure 4a). Three days after re-watering, recovery of transgenic lines was achieved in varying degrees, while the WT plants almost died (Figure 4a). Similarly, the wilted phenotype of WT after NaCl treatment for seven days was more serious than those of transgenic lines (Figure 4b). Moreover, the water loss rate of detached leaves of WT was significantly higher compared with transgenic lines, indicating that transgenic lines had better water retention capacity (Figure 4c).

**Figure 5:**
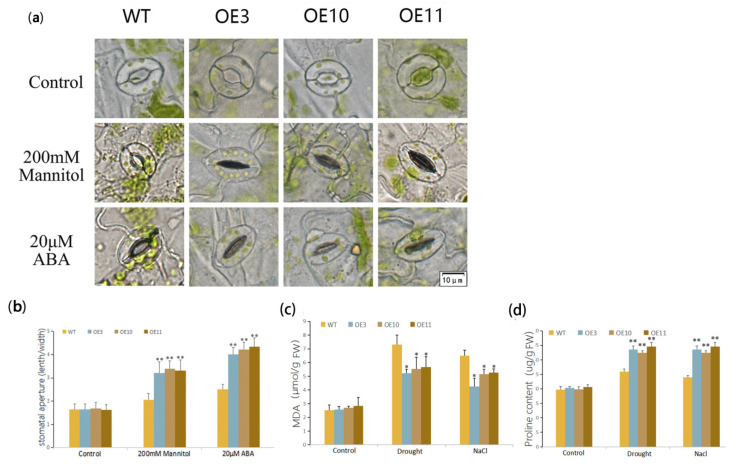
Evaluation of physiological indices responsive to stress. (**a**,**b**) Stomatal aperture of *MfWRKY70* transgenic and WT in response to 200 mM mannitol and 20 μM ABA treatments. (**c**,**d**) Malondialdehyde (MDA) content (**c**) and proline (Pro) content (**d**) were measured in OE lines and wild type (WT) plants upon drought and salinity stress at four weeks old. Data are presented as mean and SD values of three independent experiments. Asterisks indicate significant difference (* *p* < 0.05, ** *p* < 0.01, by Student’s *t*-test) comparing to WT.

We analyzed stomatal closure under stress. Treated by 200 mM mannitol and 20 μM ABA, leaves of transgenic lines had lower stomatal apertures than that of WT (Figure 5a,b) Malondialdehyde (MDA) is a well-known indicator of membrane-lipid peroxidation. Compared with the WT plants, the content of MDA increased significantly in WT and OE plants after drought and salt treatments; however, OE plants exhibited prominently lower MDA contents than WT (Figure 5c).

We also tested an important osmotic adjustment substance, proline, in WT and transgenic plants under drought and salt treatments. Proline exhibited similar abundance before stress treatment between WT and OE plants, while after drought and salt treatments, proline in all three OE lines were significantly higher than those of WT (Figure 5d).

### 3.4. Overexpression of MfWRKY70 Affected Antioxidant Metabolism Levels in Arabidopsis under Drought and Salinity Stress

**Figure 6:**
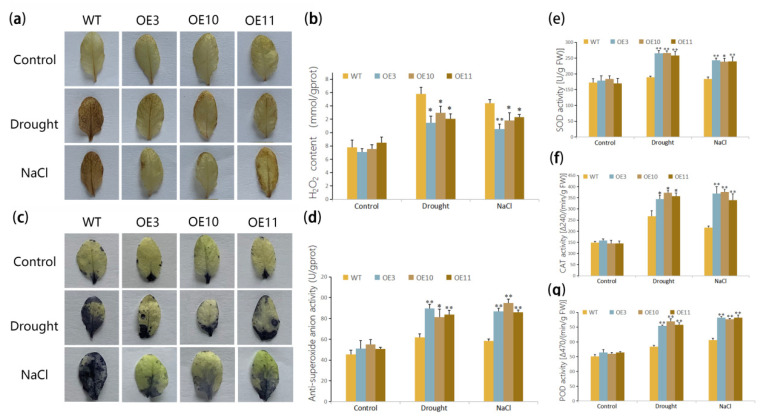
Alterations in reactive oxygen species (ROS) levels and antioxidant enzyme activities in *MfWRKY70* transgenic and WT *Arabidopsis.* (**a**,**b**) Histochemical staining by 3,3′-diaminobenzidine (DAB) (**a**) and nitroblue tetrazolium (NBT) (**b**); (**c**,**d**) H_2_O_2_ content and anti-superoxide anion activity in *MfWRKY70* transgenic and WT *Arabidopsis.* (**e**–**g**) Alterations of Activities of antioxidant enzymes superoxide dismutase (SOD) (**e**), catalase (CAT) (**f**), and peroxidase (POD) (**g**). Asterisks indicate significant difference (* *p* < 0.05, ** *p* < 0.01, by Student’s *t*-test) comparing to WT.

To determine whether antioxidant metabolism levels in *Arabidopsis* were influenced by overexpression of *MfWRKY70* under drought and salinity stress, we measured the activities of antioxidant enzymes, such as POD, SOD, and CAT, of WT and OE lines. Compared with WT plants, antioxidant enzyme activities of OE lines remarkably increased and were significantly higher after stress treatment than those of WT (Figure 6e–g). As is well known, the antioxidant enzyme plays a vital role in scavenging reactive oxygen species (ROS), such as H_2_O_2_ and O^2−^. Since antioxidant enzyme activities were changed, we then examined in situ accumulation of H_2_O_2_ and O^2−^ in leaves of WT plants and OE lines after salinity and drought stresses. By employing histochemical analysis with NBT and DAB, deeper color was stained on leaves of WT than those of transgenic lines (Figure 6a,b). Consistently, significantly lower H_2_O_2_ content and higher anti-superoxide anion activity were detected in OE lines upon salinity and drought stress compared with WT (Figure 6c,d).

### 3.5. Stress Response Genes Were Up-Regulated by Overexpression of MfWRKY70

**Figure 7:**
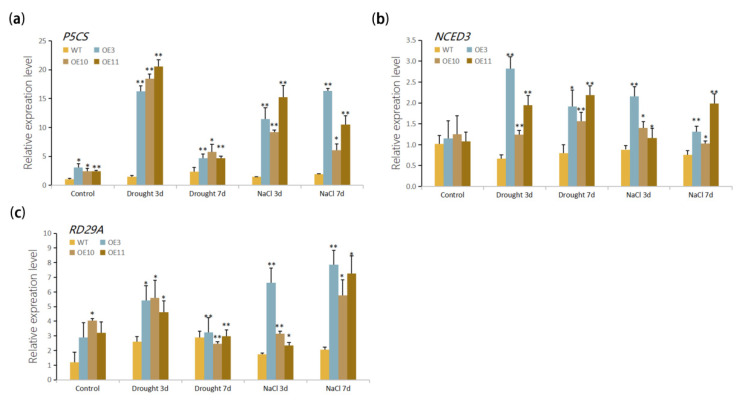
Expression levels of stress-responsive genes in *MfWRKY70* transgenic *Arabidopsis* upon drought and salinity stress tolerance. Expression levels of *P5CS* (**a**), *NCED3* (**b**), and *RD29A* (**c**) were measured by qRT-PCR in *MfWRKY70* transgenic and WT *Arabidopsis* treated by 10% PEG-6000 or 300 mM NaCl. *AtActin2* was used as an internal control. Data are presented as mean and SD values of three independent experiments. Asterisks indicate significant difference (* *p* < 0.05, ** *p* < 0.01, by Student’s *t*-test) comparing to WT.

qRT-PCR was employed to analyze the effect of *MfWRKY70* overexpression on transcript levels of stress-responsive marker genes in *Arabidopsis*. After three days and seven days of artificially simulated drought (10% PEG-6000) and salt (300 mM NaCl) treatments, transcript quantity of *AtP5CS*, *AtNCED3*, and *AtRD29A* rose more significantly in the OE lines than those of WT plants, indicating that *MfWRKY70* could directly or indirectly regulate the expression of downstream stress-responsive gene (Figure 7a–c).

## 4. Discussion

Accumulating studies indicated that plant resistance to biotic and abiotic stresses could be strengthened by overexpression of WRKY genes. For instance, ectopic expression of *FvWRKY42*, a WRKY transcription factor from *Fragaria vesca*, enhanced resistance to powdery mildew, improved osmotic stress tolerance, and increased abscisic acid sensitivity in *Arabidopsis* [5]. Moreover, heterologous expression of *TaWRKY49*, *TaWRKY92*, *TaWRKY112*, and *TaWRKY142* in wheat enhanced tolerance to abiotic and biotic stress [14]. However, few studies focusing on the role of WRKY TFs in resurrection plants, such as *M. flabellifolia,* in respect to abiotic stress response had been conducted.

In the present study, we characterized a dehydration-induced gene encoding a WRKY TF, *MfWRKY70*, from resurrection plant *M. flabellifolia.* It shared a highly conserved WRKY domain with WRKY70s from different plant species and was localized in the nucleus (Figure 1), where it may function as a transcriptional activator or inhibitor.

Roots are the most sensitive organ under abiotic stress [37,38]. O’Toole and Soemartono reported that it was most appropriate to take the length of rice roots as the morphological index for drought tolerance [39]. Some studies also demonstrated that varieties with good root growing lead to higher drought tolerance [40]. In this study, roots of all OE lines were significantly longer than those of the WT seedling, suggesting that overexpression of *MfWRKY70* significantly decreased the inhibition of root growth by osmotic (mannitol) and salinity stresses (Figure 3). Furthermore, the adult plants of OE lines also exhibited significantly better growth under long term natural drought and recovery after rewatering (Figure 4), and also exhibited better performance under salinity stress (Figure 4). These results apparently indicated that *MfWRKY70* overexpression strengthens tolerance to abiotic stress in *Arabidopsis*.

A previous study showed that group III WRKY TF AtWRKY70 and AtWRKY54 are negative regulators in osmotic stress tolerance [27]. Similarly, *PsnWRKY70* was significantly down-regulated in salt-treated *Populus simonii × Populus nigra* [41], and SlWRKY70 has a negative regulatory effect on tomato drought stress tolerance [26]. This is different from the results obtained in the present study. Thus, it is interesting to know how MfWRKY70 functions as a positive regulator of abiotic stress tolerance. Overexpression of *OsWRKY45* from *Oryza sativa Japonica,* a homologue of *AtWRKY70*, has been reported to enhance drought tolerance in *Arabidopsis* [42]. This result is similar to that of *MfWRKY70* but different from *AtWRKY70*. Two OsWRKY45 could form a dimer by exchanging their β4 and β5 strands of its DNA binding domain, then interact with W-box elements of DNA. In addition to H-bonds, salt bridges formed between two key residues of two DNA binding domains making the combination of strands hard to separate [43]. Besides, a study shows that OsWRKY45 could be activated by OsMPK6 (MAP kinase) phosphorylating one or two serine residues of its C-terminal, then binding to the W-box DNA of target gene promoters. These studies suggested that some key amino acids play a crucial role for the function of OsWRKY45 [44]. Sequence alignment showed the two serine residues are conserved in the MfWRKY70 and OsWRKY45, but are absent in AtWRKY70 (Supplementary Figure S1). Some amino acid differences among MfWRKY70 and AtWRKY70 were also found in the WRKY domains. Thus, the key amino acid sites interior the DNA binding domain or different phosphorylation sites may be causal for functional difference between AtWRKY70 and MfWRKY70.

Plants can control water loss by reducing stomatal conductance and evaporative surfaces. The stomatal operating state reflects the metabolism of plants to some extent; thus, the fast adjustment of the stomatal aperture is an important drought-tolerant feature of plants [45,46]. Our results showed that the stomatal aperture was lower in OE lines under induced by mannitol and exogenous ABA, and the water loss rates were lower during dehydration (Figure 5). These results revealed that the *MfWRKY70* OE lines have better water retention ability comparing to WT under stress.

Drought and salinity stress generally considered as osmotic stress, and the content of proline in the plant, contribute to osmotic adjustment [47,48,49]. In this study, the content of proline increased more significantly in OE lines than in WT plants, coming with the result of higher expression levels of *P5CS* under stress in transgenic *Arabidopsis,* indicating that *MfWRKY70* overexpression in plants can avoid osmotic stress by affecting the accumulation of the osmolytes.

Most abiotic stress signals induce accumulation of reactive oxygen species (ROS), particularly O^2−^ and H_2_O_2_ [50], which could cause lipid peroxidation in biomembranes [51] and lead to MDA accumulation [52]. Thus, MDA content is usually used as a parameter for the impairment of plants under abiotic stresses [53]. The antioxidant enzyme system, such as POD, SOD, and CAT enzymes, protect plants under stress treatment by keeping ROS at threshold levels and the activities of these enzymes signify the anti-stress capability of plants under abiotic stress [32,54,55]. Higher enzyme activity and lower ROS accumulation in OE lines indicated from many aspects that overexpression of *MfWRKY70* could increase POD, SOD, and CAT activities and enhance the ability of ROS scavenging under stress.

Plants respond to abiotic stress immediately at the early stage by altering the expression levels of many stress-related regulatory genes, which could regulate their downstream target genes to further react to or protect plants from stress conditions [56]. Pyrroline-5-carboxylic acid synthetase (P5CS), encoding by *AtP5CS* in *Arabidopsis*, is a rate-limiting enzyme, whose expression determines the synthetic speed of proline [57]. The *NCED3* gene plays a vital role in the regulation of ABA levels, which is the important signal responsive to abiotic stress. *RD29A* contributes to the reduction of damage by drought, salt, low temperature, and other stresses, and functions in ABA-dependent signaling pathways and the downstream target of WRKY transcription factor related to abiotic stress responses [58,59,60,61]. Up-regulation of gene expression levels of *P5CS* [62,63,64,65], *NCED3* [66,67,68], or *RD29A* usually results in increased stress tolerance. In *MfWRKY70* OE lines, the transcript amount of the three genes were upregulated compared with WT, showing that *MfWRKY70* could directly or indirectly regulate expression of *P5CS*, *NCED3*, and *RD29A* and enhance drought and salinity stresses.

Further studies on the mechanism underlying the positive role involved in plant abiotic stress response of MfWRKY70, compared to the negative role of other known WRKY70s, are needed.

## 5. Conclusions

In the present study, we cloned and characterized *MfWRKY70* of *M. flabellifolia*. Overexpression of *MfWRKY70* in *Arabidopsis* significantly enhanced abiotic stress tolerance by promoting root growth and water retention, as well as enhancing the antioxidant enzyme system and maintaining ROS homeostasis and membrane-lipid stability under stressful conditions. Moreover, the expression of stress-associated genes (*P5CS*, *NCED3* and *RD29A*) was positively regulated. These results indicated that unlike the *AtWRKY70*, *MfWRKY70* likely functions as a positive factor in abiotic stress responses.

## Data Availability

The data presented in this study are available on request from the corresponding author.

## Supplementary Materials

Supplementary materials can be found at https://www.mdpi.com/2218-273X/11/2/327/s1, Table S1: List of primers used in this study; Table S2: The GenBank accession numbers and corresponding species of some high humongous WRKYs used to construct phylogenetic tree in Figure 1. Figure S1: Sequence analysis of AtWKY70, MfWRKY70, and OsWRKY45.

## Abbreviations

TFs	transcription factors
HDTs	homoiochlorophyllous desiccation-tolerant plants
NLS	nuclear localization signal
WT	wild type
P5CS	∆-1-pyrroline-5-carboxylate synthetase
NCED	9- cis-epoxycarotenoid dioxygenase
OE	Over-Expression
DAB	3,3′-Diaminobenzidine
NBT	Nitrotetrazolium blue chloride
POD	peroxidase
SOD	superoxide dismutase
CAT	catalase
MDA	malondialdehyde
