# Supplementary material for: Dehydration-Induced WRKY Transcriptional Factor MfWRKY70 of Myrothamnus flabellifolia Enhanced Drought and Salinity Tolerance in Arabidopsis
Source: Biomolecules. 2021 Feb 22;11(2):327. doi: 10.3390/biom11020327 (PMC7926768; doi:10.3390/biom11020327)
Supplement: Supplementary file 1 [file biomolecules-11-00327-s001.pdf]

biomolecules

Supplementary

Dehydration-Induced WRKY Transcriptional Factor MfWRKY70 of *Myrothamnus flabellifolia* Enhanced Drought and Salinity Tolerance in Arabidopsis

Xiang-Ying Xiang [1,†], Jia Chen [1,†], Wen-Xin Xu [1], Jia-Rui Qiu [1], Li Song [1], Jia-Tong Wang [1], Rong Tang [1], Duoer Chen [1], Cai-Zhong Jiang [2,3] and Zhuo Huang [1,*]

[1] College of Landscape Architecture, Sichuan Agricultural University, Wenjiang 611130, China; xiangxiangying@stu.sicau.edu.cn (X.-Y.X.); chenjia@stu.sicau.edu.cn (J.C.); xuwenxin@stu.sicau.edu.cn (W.-X.X.); qiujiarui@stu.sicau.edu.cn (J.-R.Q.); songli@stu.sicau.edu.cn (L.S.); wangjiatong@stu.sicau.edu.cn (J.-T.W.); tangrong661@gmail.com (R.T.); chenduoerr@gmail.com (D.C.)

[2] Department of Plant Sciences, University of California Davis, Davis, CA 95616, USA; caizhong.jiang@usda.gov

[3] Crops Pathology and Genetics Research Unit, United States Department of Agriculture, Agricultural Research Service, Davis, CA 95616, USA

* Correspondence: huangzhuo@sicau.edu.cn; Tel.: +86-028-86290880

† These authors contributed equally to this work.

Table S1. List of primers used in this study

Usage	Primer names	Primer sequences (5′ 3′)
Clone	pGSA1403-WRKY70-F	CATGCCATGGAGTCACCTTGGTCGGA
	pGSA1403-WRKY70-R	GACTAGTTCAGAAGTCGAAAATGACATCG
Subcellular location	pHB-WRKY70-YFP-F	*ACCAGTCTCTCTCTC*CAAGCTTATGGAGTCACCTTGGTCGGA
	pHB-WRKY70-YFP-R	*GCTCACCATACTAG*TGGATCCGAAGTCGAAAATGACATCG
qRT-PCR	AtActin2-F	GGAAGGATCTGTACGGTAAC
	AtActin2-R	TGTGAACGATTCCTGGACCT
	MfWRKY70-F	TCTCTTGAAAGCCCCTCAGA
	MfWRKY70-R	CGGTGTGATGAACGAATGAC
	AtNCED3-F	CGAGCCGTGGCCTAAAGTCT
	AtNCED3-R	GCTCCGATGAATGTACCGTGAA
	AtP5CS-F	GGTGGACCAAGGGCAAGTAAGATA
	AtP5CS-R	TCGGAAACCATCTGAGAATCTTGT
	AtRD29A-F	GATAACGTTGGAGGAAGAGTCGG
	AtRD29A-R	TCCTGATTCACCTGGAAATTTCG

1 The restriction sites are under-lined and the homologous arm sequences are italicized. F and R represent the forward and reverse primers from 5'end to 3' end.

Table S2. The GenBank accession numbers and corresponding species of some highly humongous WRKYs used to construct phylogenetic tree in **Figure 1**

Genes	Accession numbers	species
PuWRKY70	QLB38140.1	*Populus ussuriensis*
PaWRKY7	XP_034913577.1	*Populus alba*
PtWRKY70	XP_002309186.3	*Populus trichocarpa*
HbWRKY70	XP_021684239.1	*Hevea brasiliensis*
MeWRKY70	XP_021619295.1	*Manihot esculenta*
JrWRKY70	XP_018814414.1	*Juglans regla*
HuWRKY70	XP_021290890.1	*Herania umbratica*
DzWRKY70	XP_022722727.1	*Durio zibethinus*
CmWRKY	ANA95961.1:20-328	*Citrus maxima*
CcWRKY70	XP_006429596.2	*Citrus clementina*
PlWRKY70	AMW90776.1	*Paeonia lactiflora*
PsWRKY	ALI57163.1	*Paeonia suffruticosa*
AcWRKY70	PSS34769.1	*Actinidia chinensis*
CsWRKY70	XP_006481203.1	*Citrus sinensis*
AtWRKY70	OAP01603.1	*Arabidopsis thaliana*

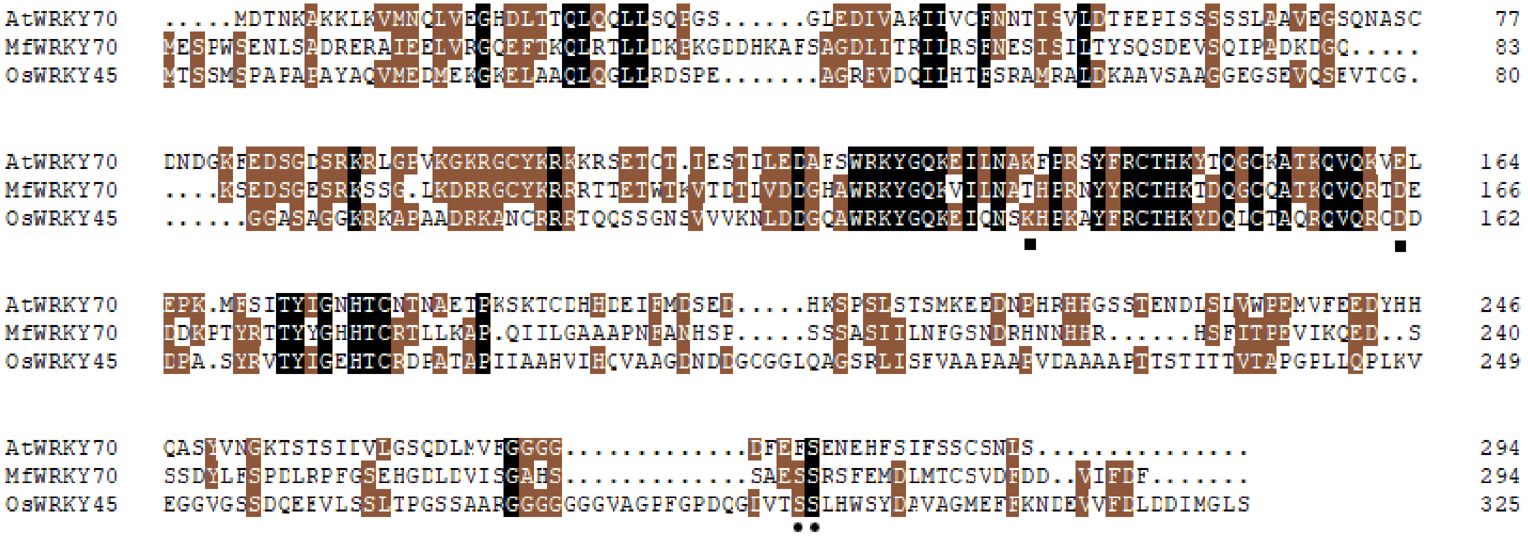

Figure S1. Sequence alignment of AtWKY70, MfWRKY70,and OsWRKY45. Two key residues about salt bridges forming of OsWRKY45 are marked by squares, two serine residues conserved in the MfWRKY70 and OsWRKY45 marked by dots.
